# Neurosarcoidosis Presented as an Isolated Brain Lesion

**DOI:** 10.7759/cureus.45837

**Published:** 2023-09-24

**Authors:** Mustafa A Khawaja, Bader E Awesat, Mohammad N Yasini, Shahed A Anzeh, Zeina R Sinnokrot, Nora I Baraghithi, Mohammed K Alayan, Abdelrhman G Abbasi, Diya M Asad, Anas N Owda

**Affiliations:** 1 Medicine, Palestine Medical Complex, Ramallah, PSE; 2 Medicine, Al-Quds University, Jerusalem, PSE; 3 School of Medicine, Al-Quds University, Jerusalem, PSE; 4 School of Medicine, Al-Quds university, Jerusalem, PSE; 5 Ophthalmology, Al-Quds University, Jerusalem, PSE; 6 Internal Medicine, Al-Quds university, Jerusalem, PSE; 7 School of Medicine, An-Najah National University, Nablus, PSE

**Keywords:** immunomodulatory therapy, pediatric neurology, cns lesions, sarcoidosis, neurosarcoidosis

## Abstract

Sarcoidosis is a multisystemic, noncaseating granulomatous disease of unknown etiology. Neurosarcoidosis (NS) is the involvement of the central nervous system (CNS) in sarcoidosis, and it occurs in approximately 5%-10% of cases. NS can present with a variety of clinical features, making diagnosis challenging. A comprehensive diagnostic approach is required to obtain a definitive diagnosis.

In this case we present a 13-year-old boy with diabetes mellitus presented with acute right-sided weakness, paresthesia, headaches, and episodes of loss of consciousness, followed by confusion and aggressive behavior. Neurological examination revealed right-sided motor and sensory deficits, as well as abnormal reflexes. Cranial imaging revealed a solitary lesion in the left centrum semi-ovale. Cerebrospinal fluid (CSF) analysis showed lymphoblastic leukocytosis, increased CSF angiotensin-converting enzyme (ACE), and a high IgG index. Extensive laboratory and imaging studies ruled out other potential etiologies.

This case presented with a unique set of clinical features, including a mass lesion effect and seizures, which are uncommon in isolated NS. The patient responded well to high-dose corticosteroid therapy, with resolution of his symptoms. Levetiracetam was used to effectively manage his seizures.

## Introduction

Sarcoidosis is a medical disorder characterized by the formation of granulomas in multiple organ systems. The exact etiology of this illness is currently unidentified [1]. Common manifestations include bilateral hilar adenopathy, pulmonary infiltration, and abnormalities in the integumentary and ocular regions [2].

Neurosarcoidosis is associated with several clinical manifestations, including cranial neuropathy, aseptic meningitis, mass lesions, encephalopathy, vasculopathy, seizures, hypothalamic-pituitary abnormalities, hydrocephalus, and myelopathy. Neurological symptoms may manifest as the initial indication in sarcoidosis patients, exceeding a prevalence of 5% [2].

The disease commonly presents in its first stages with involvement of the central nervous system (CNS), while involvement of the peripheral nervous system and skeletal muscles is more prevalent throughout the chronic phases [3]. In the absence of clinical suspicion, diagnosing neurosarcoidosis may be difficult. The occurrence of cerebral mass-like neurosarcoidosis is rarely documented in the literature [4]. The current study reports on a rare occurrence of neurosarcoidosis in a male patient; the sole proof of the sickness was a mass-like lesion inside the brain.

## Case presentation

A 13-year-old male patient with morbid obesity and a medical history of type 2 diabetes mellitus controlled with metformin was referred to our hospital in mid-June 2023 with a complaint of acute onset of right-sided weakness, paresthesia and a dull frontal and bitemporal headache. He also reported several episodes of loss of consciousness followed by confusion and aggressive behavior with no recall of the event. The patient developed multiple paroxysmal events suggestive of absence episodes. Then, the patient had several other attacks of a different type of paroxysmal event, as he describes being dizzy, after which he has some lapse in his level of consciousness but regains it rapidly with alcohol smelling, followed by a period of confusion where he becomes aggressive and has inappropriate behaviors (crying, fighting, hitting). The patient is unaware of these episodes with no recollection of the events. On physical examination, limb power was decreased on the right side (upper limb: 3/5 and lower limb: 2/5), decreased right-sided sensation, a right upward plantar reflex, and a limited upward gaze were seen. 

A cranial magnetic resonance imaging scan with contrast was promptly performed and revealed a single well-defined T2 flair hyperintense lesion noted in the left centrum semi-ovale, adjacent to the left lateral ventricle. The lesion showed more central enhancement in post-contrast T1 images, with peripherally restricted diffusion in DWI imaging. A biopsy was not performed due to refusal by the parents.

Laboratory tests, including cerebrospinal fluid (CSF) analysis, showed lymphoblastic leukocytosis with elevated albumin (38 mg/dl) and normal glucose, negative tumor cells, and negative oligoclonal bands. The level of CSF angiotensin converting enzyme (ACE) was 56.9 U/L; however, the serum ACE level was 31.1 U/L. The IgG index was reported to be high in CSF 1.39. Further investigations covered a full infectious, hormonal, and autoimmune workup, including but not limited to *Toxoplasma gondii* PCR, *Brucella*, *Mycobacterium tuberculosis*, *Treponema pallidum*, TSH, anti-Myelin-Oligodendrocyte Glycoprotein IgG (Anti-MOG IgG), Neuromyelitis Optica IgG (NMO IgG), and neurological autoimmune markers such as N-methyl-D-aspartate receptor antibody (NMDA), and all yielded negative results. 

In suspicion of neurosarcoidosis, a chest computed tomography (CT) scan was performed, which demonstrated a mild atelectatic band in the left lower lobe but no evidence of pathological infiltrates or enlarged lymph nodes. 

During the patient’s course at the hospital, he experienced several paroxysmal events characterized by confusion, followed by inappropriate behavior such as crying and fighting. Therefore, an electroencephalogram (EEG) was ordered and demonstrated focal seizures with encephalopathy and secondary generalization. 

**Figure 1:**
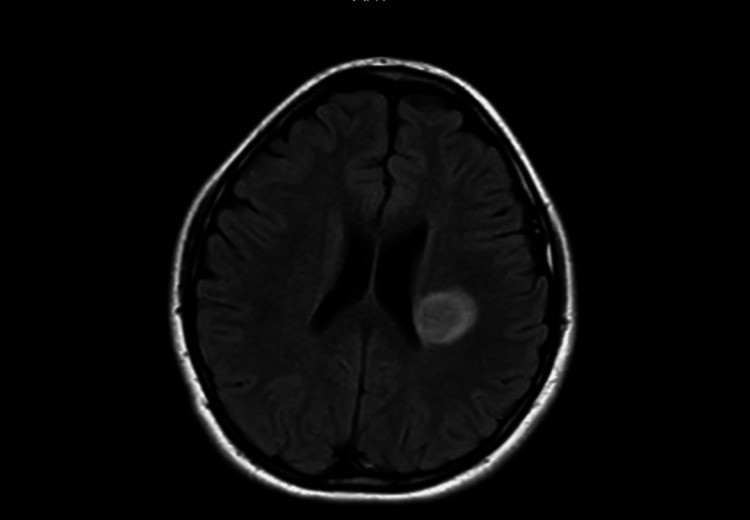
Head magnetic resonance imaging (MRI) shows a single, well-defined T2-flair hyperintense lesion noted in the left centrum semi-ovale, adjacent to the left lateral ventricle. Faintly enhanced compared to the pre-steroid MRI.

A treatment regimen for suspected neurosarcoidosis was initiated with pulse steroids (1 gram of pulsed methylprednisolone followed by rapid tapering over the course of five days) and levetiracetam 500 mg, later increased to 750 mg. Post-treatment MRI confirmed improvement of the lesion (Figure 1). Also, neurological symptoms were remarkably improved. 

## Discussion

Sarcoidosis is a multisystemic, noncaseating granulomatous disease with a nonspecific etiology. CNS involvement in sarcoidosis is uncommon, but it may affect any part of the nervous system, which leads to a difficult differential diagnosis. Central nervous system involvement, or neurosarcoidosis (NS), occurred approximately in 5%-10% of sarcoidosis cases [5]. The average age of diagnosis of NS is between 20 and 40, with a predominance of African American women in comparison with men and other ethnicities [6]. The basis of the NS diagnosis is to exclude other etiologies of granuloma formation in a patient with clinical and diagnostic pictures that suggest NS [7]. 

The definitive diagnosis for NS depends on radiological pictures with the histological conformation of a noncaseating granulomatous pattern of T lymphocytes and macrophages [5,7]. Based on the 2018 Neurosarcoidosis Consortium consensus criteria, patients are categorized into three groups according to diagnostic certainty: definite, probable, and possible NS. Definite NS is a clinical presentation that suggests NS proved by clinical manifestations, MRI, and laboratory tests after excluding other causes with CNS histological conformation. Probable NS is defined as a clinical presentation that suggests NS proved by clinical manifestations, MRI, and laboratory tests after the exclusion of other causes with systemic histological conformation. Possible NS is defined as a clinical presentation that suggests NS proved by clinical manifestations, MRI, and laboratory tests after the exclusion of other causes without any pathological conformation [8].

In our case, the patient exhibited signs of a mass lesion effect, in which he experienced a decrease in sensation, a decrease in power on the contralateral side, and seizures. This was combined with elevated levels of angiotensin-converting enzyme in the CSF sample and abnormal imaging. This patient’s clinical picture, especially with the presence of mass lesion effects and elevation of ACE levels in the CSF sample, is highly suggestive of isolated NS [9]. With the exclusion of other causes of such manifestations, the medical professionals confirmed the diagnosis of isolated NS. There was no possibility of obtaining a biopsy due to the refusal of the patient’s parents. 

The interesting manifestation experienced by our patient is the onset of seizures, which is out of the ordinary for isolated NS. According to studies, the occurrence of such seizures is only 24.5% in the pediatric population [10]. Also, the patient experienced a mass lesion effect, which is reported to occur in 5%-10% of the general population [8].

The first line of management for isolated NS is immunosuppression agents like high-dose corticosteroid therapy that our patient underwent, resulting in our patient’s remission [5]. Also, he was given levetiracetam therapy to control the episodes of seizures.

## Conclusions

Considering all this, isolated NS should be considered in patients with unexplained neurological symptoms, even when atypical presentations such as seizures and mass effects occur. Early diagnosis and immunosuppressive treatment are crucial for positive outcomes in NS patients. This case emphasizes the significance of considering NS in the differential diagnosis of CNS lesions, especially when conventional diagnostic approaches are unclear.
